# Acute Kidney Injury in Cardiogenic Shock: An Updated Narrative Review

**DOI:** 10.3390/jcdd8080088

**Published:** 2021-07-28

**Authors:** Sohrab Singh, Ardaas Kanwar, Pranathi R. Sundaragiri, Wisit Cheungpasitporn, Alexander G. Truesdell, Syed Tanveer Rab, Mandeep Singh, Saraschandra Vallabhajosyula

**Affiliations:** 1 Department of Medicine, The Brooklyn Hospital, Brooklyn, NY 11201, USA; sosingh@tbh.org; 2 Department of Medicine, University of Minnesota School of Medicine, Minneapolis, MN 55455, USA; kanwa017@umn.edu; 3 Section of Primary Care Internal Medicine, Wake Forest Baptist Health, High Point, NC 27262, USA; drpranathi99@gmail.com; 4 Division of Nephrology and Hypertension, Department of Medicine, Mayo Clinic, Rochester, MN 55905, USA; wcheungpasitporn@gmail.com; 5 Virginia Heart/Inova Heart and Vascular Institute, Falls Church, VA 22042, USA; agtruesdell@gmail.com; 6 Section of Interventional Cardiology, Division of Cardiovascular Medicine, Department of Medicine, Emory University School of Medicine, Atlanta, GA 30322, USA; srab@emory.edu; 7 Department of Cardiovascular Medicine, Mayo Clinic, Rochester, MN 55905, USA; singh.mandeep@mayo.edu; 8 Section of Cardiovascular Medicine, Department of Medicine, Wake Forest University School of Medicine, Winston-Salem, NC 27262, USA

**Keywords:** acute kidney injury, cardiogenic shock, acute myocardial infarction, critical care cardiology

## Abstract

Acute myocardial infarction with cardiogenic shock (AMI-CS) is associated with high mortality and morbidity despite advancements in cardiovascular care. AMI-CS is associated with multiorgan failure of non-cardiac organ systems. Acute kidney injury (AKI) is frequently seen in patients with AMI-CS and is associated with worse mortality and outcomes compared to those without. The pathogenesis of AMI-CS associated with AKI may involve more factors than previously understood. Early use of renal replacement therapies, management of comorbid conditions and judicious fluid administration may help improve outcomes. In this review, we seek to address the etiology, pathophysiology, management, and outcomes of AKI complicating AMI-CS.

## 1. Introduction

Despite advances in medical therapy, cardiogenic shock (CS), continues to portend a poor prognosis. Cardiogenic shock, especially related to acute myocardial infarction (AMI), is associated with a nearly 50% in-hospital mortality and a high rate of readmissions for cardiac and non-cardiac reasons [1]. In addition to cardiac etiologies for decompensation, non-cardiac organ failure plays an important factor in determining outcomes [1,2,3]. In a study analyzing the cause of readmissions in patients with AMI and CS, the majority of the causes for 30-day readmission rates in these patients were non-cardiac, out of which renal causes comprised 4.8% [4]. Acute kidney injury (AKI) (defined as ≥0.3 mg/dL or ≥50% increase in creatinine from baseline value) as per *The Kidney Disease: Improving Global Outcomes (KDIGO)* guidelines, is frequently seen in CS, and strongly associated with increased mortality [5]. AKI is one of the strongest predictors of in-hospital mortality in patients with ST-elevated myocardial infarction (STEMI) complicated by CS and associated with a poor prognosis [6]. It is understood that cardiorenal syndrome and organ hypoperfusion are the leading mechanisms of AKI; however, multiple other mechanisms may be responsible. In this review, we seek to address the etiology, pathophysiology, management, and outcomes of AKI complicating AMI-CS.

## 2. Epidemiology

Temporal trends have demonstrated a steady increase in AKI complicating AMI [2]. The incidence of AMI as a cause of CS has declined, whereas its association with chronic renal failure (+3.6%) and RRT (renal replacement therapy) (+12.4%) has increased from 1997 to 2009 [7]. The prevalence of AKI ranges from 25% to 33% of patients with acute decompensated HF [8]. In prior work by our group, utilizing the National Inpatient Sample from 2000–2014 evaluating temporal trends of AKI in AMI-CS, 35.3% of patients developed AKI and 3.4% required hemodialysis (AKI-D). A steady increase in the incidence of AKI and AKI-D was also noted [2]. The FRENSHOCK (French Observatory on the Management of Cardiogenic Shock in 2016) study noted a preponderance of non-ischemic causes for CS (60%) compared with the previous CARDSHOCK study where ischemic causes were more prevalent [9]. Women carried higher mortality compared with men (1.16 times) as depicted in a 15-year study evaluating patients with AKI complicating CS [10]. Patients with mechanical complications (papillary muscle rupture, ventricular septal defect, and free wall rupture) also had higher rates of AKI (41.1% vs. 12.7%; OR: 4.32; 95% CI: 4.13 to 4.51; *p* < 0.001) and hemodialysis (6.1% vs. 1.0%; OR: 4.45; 95% CI: 4.08 to 4.85; *p* < 0.001) compared with those without mechanical complications. It is noteworthy that 49.5% of these patients were treated with mechanical circulatory support (MCS) such as intra-aortic balloon pump (IABP) (42.1% in 2003 and 36.1% in 2015), percutaneous ventricular assist devices (pVADs) (0% in 2003 to 7.6% in 2015) and extracorporeal membrane oxygenation (ECMO) (0.5% in 2003 to 8.4% in 2015) [11]. This also calls to attention the interplay of factors between MCS use and AKI.

## 3. Pathogenesis

**Figure 1:**
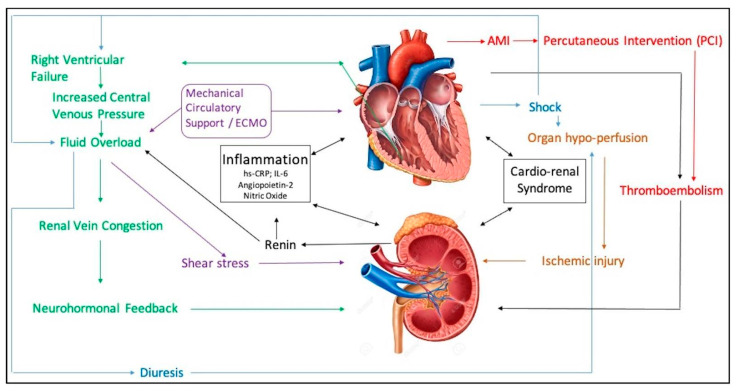
Pathogenesis of acute kidney injury in acute myocardial infarction with cardiogenic shock.

The abrupt decrease in blood flow and end-organ hypoperfusion leading to oliguria in patients with CS is well described [12]; however, additional mechanisms such as inflammation, systemic and venous congestion, right ventricular failure, mechanical circulatory support, thromboembolism, and contrast nephropathy that play pivotal roles in worsening renal failure in AMI-CS have not been well elucidated (Table 1 and Figure 1) [13].

### 3.1. Role of Inflammation

#### 3.1.1. C-Reactive Protein

The role of inflammation in CS is well described; however, the correlation of AKI with an increase in levels of inflammatory markers, such as high sensitivity C-reactive protein (hs-CRP), in AMI indicates a possible role of inflammation with renal tubular epithelium representing a major site of cell injury and death [14,15]. In addition, structural monomers of CRP are hypothesized to cause the activation of platelets, leukocytes, endothelial cells, and complement, thus mediating ischemia and reperfusion injury to the kidney [16]. In vitro, CRP was shown to activate the mitogen-activated protein kinase (MAPK) pathway that further recruited leukocytes into inflammatory sites in human renal distal tubular cells in a dose-dependent manner [17]. CRP is also postulated to promote the CD32-Smad3-p27-driven inhibition of the CDK2/cyclin E complex that impairs tubular epithelial cell regeneration, hence contributing to AKI [18]. In a prospective observational study, hs-CRP levels were higher in AKI patients vs. those without (45 ± 87 vs. 16 ± 41 mg/L; *p* < 0.0001). Shacham et al. prospectively investigated 562 STEMI patients undergoing primary percutaneous intervention (PCI) and reported that those with hs-CRP levels >9 mg/L at hospital admission had higher AKI (17% vs. 6%) and 30-day mortality (11% vs. 1%) rates than patients with hs-CRP levels below this limit and hs-CRP > 9 mg/L at admission in that study was an independent predictor for AKI [19].

#### 3.1.2. Angiopoietin and Interleukin

Inflammation also plays a pivotal role in patients with AMI-CS complicated by AKI. As evidenced in the IABP SHOCK II (Intra-Aortic Balloon Pump in Cardiogenic Shock) trial, higher levels of angiopoietin-2 (regulator of vascular barrier function) were independently associated with worse short and long-term outcomes in patients with CS [20]. Interleukin-6 (IL-6), which is known to be an independent prognostic predictor of 30-day mortality in patients with AMI-CS, also correlates with increased incidence of AKI and a higher need for vasopressors in this cohort [21,22]. In another study, patients with CS and multiorgan failure exhibited similar magnitudes of IL-6 levels to those found in patients with septic shock [23]. These mediators of inflammation, as described above, impair myocardial function and accelerate heart failure (Braunwald, 2008) [24]. They also activate the vascular endothelium to promote inflammation, oxidation, and vasoconstriction (Gimbrone et al., 2000) [25].

#### 3.1.3. Nitric Oxide

Nitric oxide pathway biomarkers are also associated with the occurrence of AKI in STEMI patients [26]. End organ hypoperfusion, leading to local nitric oxide pathway, is a well-established factor contributing to AKI.

#### 3.1.4. Role of Novel Biomarkers

Traditional biomarkers such as serum creatinine (sCr) and urine output have often delayed the detection of AKI [27]. Serum cystatin-c has improved the estimation of eGFR irrespective of muscle mass and dietary intake. A higher cystatin C value at discontinuation of RRT is an independent predictor of chronic dialysis in patients receiving RRT [28]. However, novel biomarkers that can identify AKI before the injury becomes irreversible are now emerging [29,30,31]. In the Prevention of AKI trial, timely intervention, guided by the urinary concentration of stress biomarkers, such as tissue inhibitor of metalloproteinases 2 (TIMP-2) and insulinlike growth factor binding protein 7 (IGFBP7) (level of TIMP-2 X IGFBP7 of at least 0.3 ng/mL^2^/1000) after cardiac surgery, led to a 17% reduction in AKI [32,33]. In the same cohort, levels less than 0.3 ng/mL^2^/1000 did not have increased progression to more severe AKI [34]. In a study of 733 patients undergoing cardiac surgery, preoperative urinary concentration of dickkopf-3, a urinary cytokine and tubular stress biomarker, successfully predicted postoperative AKI with an area under the receiver operating characteristic curve (AUROC) of 0.78 [29]. A novel biomarker reflecting kidney damage—urine neutrophil gelatinase–associated lipocalin (uNGAL)—was found to be superior to sCr in predicting the severity and persistence of AKI. A study of 178 children demonstrated that elevated uNGAL concentration, even without an increase in sCr, was associated with an almost four-fold increase in the risk of AKI (all stages). Similarly, in patients receiving mechanical ventilation, sCr when combined with uNGAL and serum cystatin-c, was found to be a better predictor for RRT initiation when compared with those without an uNGAL and sCr increase (AUROC = 0.80) [35]. In a recent analysis of the 154 patients with AKI-CS, from the prospective CardShock study, high baseline levels of markers of AKI, such as plasma proenkephalin (P-PENK) > 105.7 pmol/L and neutrophil gelatinase-associated lipocalin (P-NGAL) > 151 ng/mL, at 24 h, were found to be independent predictors of 90-day mortality. However, it should be noted that these biomarkers are not recommended for predicting the occurrence of AKI before a nephrotoxic insult occurs. Further research is needed for employing biomarkers that risk stratify patients prone to developing AKI [27,36].

### 3.2. Role of Right Ventricular Failure/Renal Vein Congestion

It is known that lower arterial pressure in patients with CS leading to end organ hypoperfusion is associated with both AKI incidence and severity [5]. Cardiorenal syndrome resulting in AKI has been well described as a complication of CS and is an independent driver of mortality in patients with STEMI [37]. However, the role of RV failure is less well understood in the pathogenesis of AKI complicating AMI-CS. As demonstrated by Van den Akker et al., patients with AKI have higher levels of central venous pressure (CVP) that aids in the development of AKI in patients with shock [38]. In the ESCAPE (Evaluation Study of Congestive Heart Failure and Pulmonary Artery Catheterization Effectiveness) trial, right atrial pressure was the only hemodynamic parameter that correlated with estimated glomerular filtration rate (eGFR) [39]. This was also evidenced by another study analyzing factors affecting hemodynamics post-PCI, where right atrial pressures were inversely associated with eGFR within 1 week after PCI (β = −1.66; 95% CI −3.06 to −0.25). In the same study, however, the volume of contrast used was not associated with eGFR after catheterization [39].

Analysis of the Mayo Clinic Rochester Cardiac intensive care unit (CICU), from 2007 through 2018 revealed that heart failure (HF) has overtaken acute coronary syndrome (ACS) as the most common cause of CS in the CICU [40]. Hanberg et al. demonstrated that overall cardiac index (CI) or change in CI leading to organ hypoperfusion is not the primary driver for renal dysfunction in patients hospitalized for HF. It may play a role in less severe forms of acute HF, but not as much in CS [41]. The study by Matejka et al. found HF to be the strongest predictor of AKI, and that patients with AKI had a higher incidence of CS (44% vs. 5%) [42]. Venous congestion, as evidenced by higher CVP (>8 mm Hg), is a crucial hemodynamic factor determining worsening renal function in patients with advanced decompensated HF [43]. In a large study at a tertiary center in the Netherlands, CVP was an independent predictor of reduced survival (hazard ratio: 1.03 per mm Hg increase in CVP, 95% confidence interval: 1.01 to 1.05, *p* = 0.0032) [44].

It is postulated that RV dysfunction can lead to increased CVP leading to increased back-pressure and renal vein congestion. This is supported by animal studies in which increasing renal venous pressure, mediated by a decreased renal perfusion leads to a reduction in glomerular filtration [19,45]. Local hypertension in the glomerulus and resultant tubulointerstitial hypoxia cause loss of glomerular integrity and tubular damage giving rise to cardiorenal syndrome [46,47]. Secondarily, venous congestion also stimulates the release of proinflammatory cytokines such as TNF (tumor necrosis factor) and IL-6.

Renin is another hormone secreted during this proinflammatory state that sets up a vicious cycle of fluid retention, leading to further venous congestion, impaired renal function and worsening of cardiac function (Sánchez-Lozada et al.) [48]. Lastly, there has been an increase in the use of pulmonary artery pulsatility index (PAPi), defined as the ratio of pulmonary artery pulse pressure to right atrial pressure, as a more composite marker of RV dysfunction [49]. Though PAPi has not been specifically investigated in the CS population, lower pre-operative PAPi in heart transplant recipients has been associated with higher rates of post-operative AKI [50]. This may allude to a systematic remodeling of the renal and systemic veins that continue to impact renal function independent of cardiac function.

### 3.3. Role of Thromboembolism

The atheroembolic phenomenon resulting from endovascular procedures causes AKI in addition to contrast-induced nephropathy (CIN). In a study, AKI continued to occur in high-risk STEMI patients, despite the preventive measures (use of non-ionic, water-soluble iodinated contrast media), indicating that various other mechanisms may be at play [42].

### 3.4. Contrast-Induced AKI

Older age may independently contribute to CI-AKI (OR:1.23 95% CI: 1.16–1.31, *p* < 0.001) as depicted in a recent pooled data analysis of the FRASER and HULK studies [51]. Contrast-induced AKI develops more frequently in patients with CS than those without (7% vs. 1%, *p* < 0.001). The volume of contrast media used also seemed to play no role when adjusted for body mass index (BMI) (*p* = 0.079) [52]. Additionally, contrast media-dose to estimated glomerular filtration rate ratio is a better marker to assess the risk of CIN; however, the risk of CIN only marginally increases with decreasing eGFR [53].

### 3.5. Role of Mechanical Circulatory Support

Novel MCS devices, such as Impella^®^, TandemHeart^®^, and ECMO have positively impacted the management of CS. The use of ECMO, along with concomitant procedures (percutaneous coronary intervention, intra-aortic balloon pump, and percutaneous LVAD) has steadily risen over the years. However, they are not devoid of complications [54,55]. AKI is known to occur in 70–85% of patients receiving ECMO [56]. A meta-analysis studying the complications of ECMO found that AKI occurred in 55.6% (35.5–74.0%) of patients [57]. A proposed mechanism is loss of pulsatile flow resulting in shear stress on the renal epithelium [57]. Margolis et al. showed that positive fluid balance is strongly associated with higher stage of AKI (52% vs. 13%) and lower rate of renal recovery (29% vs. 75%) in patients with AMI-CS. Positive fluid balance in ECMO is an independent predictor of 90-day mortality in patients being treated for refractory HF [58]. In an experimental study using a swine model to estimate microcirculation of the heart and kidney in pulsatile and nonpulsatile-assisted circulation, the results suggested that pulsatile assist was more effective than nonpulsatile assist for microcirculation after CS to avoid the deterioration of major organ functions [59]. ECMO, along with the use of vasopressors/inotropes and their frequent adjustments, causes hemodynamic instability and fluctuations in renal blood flow. This exacerbates ischemia/reperfusion-associated AKI [60]. Impeller-related hemolysis with axial flow devices, such as Impella, is also increasingly being recognized [61]. Additionally, among AMI-CS patients receiving MCS, AKI is known to more commonly occur with pLVAD compared with IABP (55.4% vs. 39.1%; *p* < 0.001) [62].

Abadeer et al. postulated that short-term MCS with ECMO or ventricular assist devices (VAD) may also contribute to renal damage through hemoglobinuria-induced renal injury. This is caused by blood exposure to artificial surfaces, resulting in hemolysis in the extracorporeal circuit [63]. Another possibility is excessive fluid overload during ECMO exacerbating the renal vein congestion by mechanisms as described above. Furthermore, bleeding and thromboembolism associated with short term-MCS could also cause AKI by mechanisms that are yet to be explored [56].

## 4. Outcomes

### 4.1. In-Hospital Outcomes

The current literature demonstrates that higher mortality, length of stay and hospital costs are associated in AMI-related CS complicated by AKI. In several studies, AKI has been noted as an independent predictor of in-hospital mortality in AMI-CS. Those requiring hemodialysis (HD) or RRT carried worse mortality rates (75.74% vs. 51.58%) and length of stay (21.4 vs. 14.4 days) [64,65]. In a Danish cohort where patients with AMI-CS complicated by AKI received RRT, those who did receive RRT had higher in-hospital mortality compared with those who did not (62% vs. 36%) [66]. Notable sex disparities also exist in these patients [67]. A study of the National Inpatient Sample from 2000–2014 of patients with AMI-CS complicated by AKI revealed women were older, more often of non-White race, and had more co-morbidities compared with men. They had higher in-hospital mortality, and were less often discharged to home compared with men [10]. These data were upheld in another research paper where female sex, regardless of age, was found to be an independent predictor of in-hospital mortality (23.0% vs. 21.7%; OR:1.11 [95% CI, 1.07–1.16]; *p* < 0.001) for patients aged <55 years and (OR:1.05; 95% CI, 1.02–1.08; *p* < 0.001) aged > 75 years [68,69] In a previous study by our group, the patients with AKI-ND and AKI-D, as compared to those without AKI, were older (71 ± 13 and 69 ± 12 years vs. 68 ± 13), male (64% and 66% vs. 59%), with higher rates of diabetes (5% and 5% vs. 4%), HF (64% and 70% vs. 50%,), chronic kidney disease (23% and 36% vs. 7%), presented with non-ST-segment-elevation AMI-CS (40% and 47% vs. 27%), and received less frequent coronary angiography (63% and 65% vs. 70%) and PCI (42% and 37% vs. 51%) (all *p* < 0.001). AKI was also associated with higher incidence of non-cardiac organ failure, cardiac arrest, and the use of invasive hemodynamic monitoring, mechanical ventilation, and MCS [2]. In STEMI-CS patients undergoing IABP support and PCI, AKI was noted to be the strongest independent predictor of in-hospital mortality (RR: 12.3, 95% CI 1.78 to 84.9; *p* < 0.001). Patients with AKI had a longer hospital stay and higher mortality rate (50% vs. 2.2%; *p* < 0.001) than those without. Older age > 75 years, left ventricular ejection fraction ≤ 40%, and the use of mechanical ventilation were found to be independent predictors of AKI [70].

### 4.2. Long-Term Outcomes

Studies depict that AKI is not just limited to in-hospital mortality. It is shown to be an independent prognostic factor for long-term mortality, among patients with STEMI-CS, and treated with primary PCI (HR: 2.207; 95% CI: 1.150–4.739) [71]. AKI is an independent risk factor for prolonged hospitalization, need for RRT, readmission, increased stroke risk, and mortality [8]. In a study analyzing the impact of CKD in patients with left main coronary artery as a culprit undergoing PCI, eGFR emerged as a predictor of 1-year mortality, (OR = 0.97, 95% CI: 0.95–0.99, *p* = 0.005). The patients with renal failure were older, had more diabetes, and more frequently experienced AMI [72]. AMI-associated AKI is associated with more than a three-fold increase in early mortality and more than two-fold in long-term mortality, as demonstrated by Pickering et al. [72]. Patients with AMI-AKI tend to have a higher long-term mortality (57.3 vs. 20.6%; *p* < 0.0001) even if treated with PCI. Even a slight increase in serum creatinine is associated with a progressive increase in long-term mortality, as demonstrated by a study at a large institution [11]. Renal failure also transcended as an independent mortality predictor in patients with out-of-hospital cardiac arrest (OHCA) complicating a STEMI that was immediately treated with PCI (64.3% vs. 30.0%, *p* = 0.004). These patients also had a longer duration of stay in the ICU and required mechanical ventilation for more than forty-eight hours [63]. In a prospective German study that followed patients with AMI requiring mechanical ventilation, 19% of the 458 patients developed AKI [73]. In patients receiving emergent coronary artery bypass grafting (CABG) for AMI, even though in-hospital mortality declined over the years, an increase in multiorgan failure, particularly renal failure, was noted (24.7% in 2012–2017 vs. 9.8% in 2000–2005; *p* < 0.001) [74]. Abadeer et al. have previously demonstrated severe AKI (stage 3) as a predictor of long-term mortality (HR, 1.54; CI 1.10–2.14; *p* = 0.011) in patients with short-term MCS for CS [63]. In prior work from our group, patients with CS and AKI-HD tended to have higher comorbidity burdens and were also more likely to receive MCS devices compared with those without AKI-HD. The association with AKI-HD also shows significantly higher in-hospital mortality (75.74% vs. 51.58%, *p* < 0.001), use of MCS (24.0% vs. 19.3%, *p* < 0.001), length of stay (21.4 vs14.4 days, *p* < 0.001) and hospitalization cost (USD 80,406 vs. USD 52,833, *p* < 0.0001) [75]. In data from the Bremen-STEMI registry, AKI was a significant predictor of an increased 1-year mortality (OR 3.6; 95% CI 1.9–7.0) in STEMI-patients with CS [76]. In patients with AMI who underwent PCI after cardiac arrest, AKI was the strongest predictor of 30-day mortality (adjusted OR 6.98; 95% CI 3.42 to 14.23; *p* < 0.0001). The other significant predictors were bleeding, CS, contrast volume-to-glomerular filtration rate ratio, and female sex [8].

Mechanical complications tend to occur more in STEMI compared with NSTEMI hospitalizations (0.27% vs. 0.06%), and were associated with higher in-hospital mortality (42.4% vs. 18.0%) [76]. In patients who develop AKI within 24 h after the onset of CS, mortality was as high as (87% vs. 53%) compared to those without [63], RRT also was associated with higher mortality in patients with AMI [77]. In a large retrospective cohort study of AMI-CS using the national inpatient sample by our group, multiorgan failure was associated with higher mortality in AMI-CS [1]. Mechanical complications tend to occur more often in STEMI compared with NSTEMI hospitalizations (0.27% vs. 0.06%), and are associated with higher in-hospital mortality (42.4% vs. 18.0%) [77]. In patients who develop AKI within 24 h after the onset of CS, mortality was as high as 87%, compared to 50% in those without AKI [64]. RRT was also associated with higher mortality in patients with AMI [78]. In a large retrospective cohort study of AMI-CS using the national inpatient sample by our group, multiorgan failure was associated with higher mortality in AMI-CS [1].

Worsening renal failure (defined as 25% reduction in GFR from admission within 7 days of hospitalization) in patients with ACS is an independent predictor of in-hospital mortality (adjusted odds ratio 28.02, 95% CI 13.2–60.28, *p* < 0.0001) [79]. The cohort in this study was older, had more cardiovascular risk factors, more likely to be female, with a history of vascular disease, and presented with more NSTEMI than patients without WRF (39.5% vs. 32.8%; *p* = 0.042).

## 5. Management

### 5.1. Cardiac Management

AKI from AMI-CS represents a form of cardiorenal syndrome type-1, wherein primary acute cardiac failure is associated with acute renal failure [80]. In such patients, management of the primary cardiac disorder with close understanding of the implications on the management of renal function is crucial. A full review of the invasive and intensive care management of AMI-CS is beyond the scope of this review, and has been highlighted by multiple high quality reviews previously [81,82]. In brief, emergent revascularization in both STEMI and NSTEMI forms the cornerstone of management in this population. Recent data from the CULPRIT-SHOCK (culprit lesion only PCI versus multivessel PCI in cardiogenic shock) trial has specifically demonstrated the impact of culprit-lesion only PCI on renal outcomes [83]. In 706 patients with AMI-CS, the composite outcome of death and use of RRT was lower in the culprit-only PCI group (45.9% vs. 55.4%; relative risk ratio 0.83 [95% CI 0.71–0.96]; *p* = 0.01). Though baseline creatinine values were similar between groups (1.17 mg/dL vs. 1.20 mg/dL), the end point of RRT use trended towards significance (11.6% (culprit-only PCI) versus 16.4% (multivessel PCI); relative risk 0.71 [95% CI 0.49–1.03]; *p* = 0.07) [83]. A novel percutaneous mechanical circulatory support (pMCS) device currently under trial, specifically designed to address renal perfusion (albeit being tested in a chronic heart failure population), is the Aortix™. It acts by increasing the overall velocity and aortic flow to manage renal insufficiency [84].

The optimal method of pharmacological and MCS in these patients for the prevention of AKI is still not fully understood. The SOAP-II (Sepsis Occurrence in Acutely Ill Patient) trial that evaluated all comers with circulatory shock did not demonstrate any differences in RRT-free days (within 28-days) between norepinephrine and dopamine [85]. Similarly, the OptimaCC (Study Comparing the Efficacy and Tolerability of Epinephrine and Norepinephrine in Cardiogenic Shock) trial, did not show any differences in RRT use between epinephrine and dopamine an all-comer CS population [86]. Current best practice guidelines recommend norepinephrine as the first-line vasopressor for the management of CS with the addition of other pharmacological or mechanical support agents as indicated [82].

### 5.2. Renal Management

Commonly, lactate levels are used to monitor organ perfusion; however, patients with a lower base excess and bicarbonate levels than lactate levels are a better marker for poor prognostic value. Lower serum bicarbonate is independently associated with higher mortality in patients with ischemic CS in the ICU setting [87,88]. Fluid administration has been a cornerstone of management in patients with AKI; however, continued fluid administration resulting in positive fluid balance, especially in patients with AMI-CS, has not been shown to improve renal outcomes and may even worsen overall prognosis [89]. Instead, volume-directed fluid therapy, guided by careful volumetric and arterial waveform derived variables, as compared with conventional monitoring, may decrease the incidence of AKI (4.3% vs. 28.6%, *p* = 0.03) in patients with CS after cardiac arrest, as demonstrated by Adler et al. [90]. In patients with AKI-CS, early initiation of renal replacement therapeutic strategy, after cardiac surgery, based on long-lasting continuous veno-venous hemofiltration (CVVH) could improve patients’ outcomes [91]. Early and higher dose use of continuous renal replacement therapy (CRRT), resulted in lesser all-cause mortality rates (45.4% vs. 73.3%) (*p* = 0.002) and in-hospital mortality rates (61.8% vs. 82.2% (*p* = 0.02)) compared with those who received lower doses of CVVH [92]. Diuretics such as furosemide, although they may help relieve pulmonary edema in CS, are not useful when complicated by AKI [93]. In patients with AMI who have recovered from their CS, the presence of AKI precludes the use of guideline-directed medical therapy such as angiotensin-converting enzyme-inhibitors, angiotensin receptor blockers, and aldosterone inhibitors [94]. As inflammation is one of the main pathogenetic factors in AKI complicating AMI-CS, targeting CD32-Smad3-p27 as activated by CRP, may offer a new treatment approach for AKI [18].

### 5.3. Supportive Therapies

In addition to cardiac and renal management, the management of other failing organs has a crucial role in prevention and mitigation of AKI. RV protection, with the use of low positive end-expiratory pressure on the mechanical ventilator, the prevention of hypoxemic pulmonary vasoconstriction and minimizing fluid overload all contribute to improvement in cardiorenal interactions [95]. The management of iatrogenic infections, including the development of concomitant sepsis and septic shock in this population is crucial since sepsis is an independent predictor of renal failure in this population [96,97]. In addition, meticulous avoidance of nephrotoxic agents, such as antimicrobial drugs (particularly aminoglycosides), nonsteroidal anti-inflammatory drugs, and iodinated radiocontrast is crucial to lower the incidence of AKI [98].

## 6. Future Directions

Recognizing AKI in a time sensitive manner may help reduce economic burden, readmission rates and improve in-hospital mortality [4]. Clinicians must be aware of the judicious use of diuretics while taking into consideration other factors at play, such as RV failure, in addition to end organ perfusion, while managing AKI in AMI-related CS. The monitoring of inflammatory markers, such as IL-6, especially in AMI-CS, proclaims better estimation of prognosis. The use of pulmonary artery catheter (PAC) in AMI-CS has become ancillary owing to its association with worse outcomes [99]. The early use of CRRT, revascularization and judicious fluid resuscitation without preventing fluid overload, may benefit patients and improve outcomes. Furthermore, the role of interdisciplinary teams in AMI-CS may help improve outcomes in this critically ill population [100]. Further trials are warranted in this critically ill population to aid in the development of best practices for AKI management in AMI-CS.

## Figures and Tables

**Table 1:** Acute kidney injury in acute myocardial infarction-cardiogenic shock.

Mechanism	Markers	Pathogenesis
Inflammation	hs-CRP, IL-6, angiopoietin-2, nitric oxide	MAPK → impairs tubular epithelial cell regeneration → impairs tubular epithelial cell regeneration pathway Capillary leakage
Right ventricular failure	Central venous pressure	Renal vein congestion → neurohormonal feedback → tubular cell injury → inflammation Cardio-renal syndrome
Mechanical circulatory support	NT-ProBNP	Loss of pulsatile flow → shear stress Fluid overload → renal vein congestion Hemolysis in extracorporeal circuit Impella (intracorporeal motor)-related hemolysis
Decreased cardiac index	Serum bicarbonate, lactate	Organ hypoperfusion leading to ischemic injury
Thromboembolism/contrast-induced nephropathy	Contrast medium dose-to-eGFR ratio	Cell-mediated injury Thromboembolic shower during PCI

Abbreviations: hs-CRP: high-sensitivity C-reactive protein; IL: interleukin; MAPK: mitogen-activated protein kinase; NT-proBNP: N-terminal pro-B-type natriuretic peptide; PCI: percutaneous coronary intervention.

## Abbreviations

AMI	acute myocardial infarction
AKI	acute kidney injury
CS	cardiogenic shock
CI	cardiac index
CRRT	continuous renal replacement therapy
CVVH	continuous veno-venous hemofiltration
CVP	central venous pressure
ECMO	extracorporeal membrane oxygenation
HF	heart failure
hs-CRP	high sensitivity C-reactive protein
IABP	intra-aortic balloon pump
MCS	mechanical circulatory support
MAPK	mitogen-activated protein kinase
PMR	papillary muscle rupture
pVADs	percutaneous ventricular assist devices
PCI	primary percutaneous intervention
RRT	renal replacement therapy
RV	right ventricle/right ventricular
STEMI	ST-segment-elevation myocardial infarction
VSD	ventricular septal defect
